# Dye-Sensitized Solar Cell Based on TiO_2_ Anode Thin Film with Three-Dimensional Web-like Structure

**DOI:** 10.3390/ma15175875

**Published:** 2022-08-25

**Authors:** Yang Liu, Jinzhu Chen, Zhihua Tian, Jianxi Yao

**Affiliations:** 1 School of Nuclear Science and Engineering, North China Electric Power University, Beijing 102206, China; 2 State Key Laboratory of Nuclear Resources and Environment, East China University of Technology, Nanchang 330013, China; 3 Renewable Energy School, North China Electric Power University, Beijing 102206, China

**Keywords:** dye-sensitized solar cell, TiO_2_ film, the barrier layer, surface-modified layer

## Abstract

TiO_2_ films with a three-dimensional web-like porous structure were prepared using the photo polymerization-induced phase separation method integrated with the pulling coating process. By adjusting the ratio of the substance in the precursor sol and the coating times, the relationships between the sol ratio, the coating times, the film structure, and the performance of the DSC were studied. The optimal film structure was found and a detailed description is given. The performance of the DSC was further improved by introducing the barrier layer and the surface-modified layer of the TiO_2_ coating. This promoted the short-circuit current density and the photoelectric conversion efficiency of the DSC, the mechanism of which was also investigated. Ultimately, the photoelectric conversion efficiency of the DSC based on the TiO_2_ anode films with a three-dimensional web-like structure was stabilized at a higher level as a result of the structural improvement.

## 1. Introduction

In the past decades, the fabrication of macroporous materials has seen significant advances due to their potential applications as photonic crystals [1], filtration and separation agents [2,3], and anode materials in Li-ion batteries [4,5]. Among these materials, macroporous titanium dioxide film is particularly interesting, because it is a wide-band-gap semiconductor with a high dielectric constant, high refractive index, and good resistance to chemical attack. Moreover, it is widely used in pigments, solar cells, humidity and gas sensors, photocatalytic degradation, the selective reduction of NOx, purification, odor control, and sterilization [6,7,8,9,10,11,12]. The properties and performance of TiO_2_ film are highly dependent on its morphology and preparation process. Many recent efforts have been devoted to the controlled fabrication of macroporous TiO_2_ films, such as those based on powder sintering [11], self-assembly of block copolymers [13], selective etching [14], sol-gel [15,16,17,18,19,20], and the extraction–pyrolytic method [21]. In the case of the sol-gel method, controlling the porosity in the upper limit of the macroporous scale is achieved by using larger colloidal templates, such as latex or silica nanometric beads and emulsion or foam templating [16,17,18], or by using controlled phase separation in the presence of solvent mixtures and polymer [22,23,24,25,26,27,28,29,30,31,32,33]. The combination of different templates permits the synthesis of macroporous TiO_2_ films with different morphologies.

Incorporating organic polymers such as poly (ethylene glycol) (PEG) [22], polypeptides [23], or nonylphenyl ether (NPE-20) [25] in sol-gel processes is a simple strategy to produce macroporous oxide films. Titania macroporous thin films with an enhanced surface area were produced with the aim of improving their photocatalytic properties. Nakanishi and co-workers made a pioneering exploration of the effect of the synthesis conditions on film morphology with the addition of PEG [26], and a comprehensive study of each variable was performed by Kajihara and Yao [26,27,28,29,30,31]. From this earlier work, it was proposed that the PEG templating agent forms a stable complex with Ti-oxo species and that the macropores are generated by the phase separation of the solvent. The phase separation process is induced by the strong interactions between the inorganic oligomers and the organic polymer, which repel the solvent. Yoko et al. prepared macroporous polycrystalline TiO_2_ films with a two-dimensional spinodal phase-separated structure using NPE-20 [25,32,33]. From these studies, it was proved that the polycondensation reaction proceeds as solvent evaporates from the surface of the gel film after the dip-coating operation, and the compatibility between the TiO_2_-rich phase and the polymer-rich phase decreases, leading to the initiation of spinodal phase separation.

Despite the insights gained regarding the synthesis mechanism of titania macroporous films, some aspects still have to be considered. Compared to bulk systems, an overlap of solvent evaporation and polycondensation stages, a rapid deposition process, and a high sensitivity to environmental conditions are characteristic in the film systems. Controlled macropores of various sizes and distributions can be fabricated as a result of the interaction between the polymerizable metalxane oligomer, solvent mixture, and incorporated polymer. Generally, most of the attempts to prepare such films only resulted in two-dimensional structures with a lower surface area. In addition, the morphology of the prepared TiO_2_ films was strongly affected by the deposition process and environmental conditions [25,26,27,28,29,30,31,32,33]. Therefore, it is very difficult to obtain a film with a homogeneous morphology and high reproducibility in a titania-alkoxide-based system. In order to improve the photocatalytic and photoelectronic performance of TiO_2_ films, it is necessary to fabricate a TiO_2_ film with a high surface area. Three-dimensional macroporous (3DM) TiO_2_ films can be viewed as a good candidate for the improvement of such properties. This kind of 3DM architecture may have interesting bulk-chemistry applications, especially for heterogeneous photocatalysis, photonic crystals, and opto-electricity conversion [34,35,36,37]. This is because they enhance the quantum yield due to an enlarged surface area and multiple scattering. In addition, they introduce a continuous pore channel that facilitates the transfer of reactant molecules [38].

The photo polymerization-induced phase separation (PIPS) method is a common method to fabricate microstructures in polymer dispersed liquid crystal films [36,37,38,39,40]. In the PIPS process, polymerization is induced by uniformly irradiating a prepolymer (monomer, LC, photoinitiator) cell of controlled thickness. At some point after the polymer starts to grow, the miscibility gap is breached, and phase separation of discrete phases occurs. Control of the irradiation intensity, component volume fractions, and temperature are typically used to control the resulting morphology (domain size, domain shape, volume fraction) of the product [36,37].

In the present study, we made an attempt to prepare TiO_2_ films with a 3DM structure using the PIPS method. A low molecular titanium alkoxide was mixed with a reactive monomer and a polymerization initiator. The polymerization of the photomonomer was then initiated by UV irradiation. In the PIPS process, phase separation and polymerization simultaneously occurred. Phase separation occurred as the molecular weight and crosslink density of the growing polymer chains increased. Similar to the LC system, the morphology of the as-prepared TiO_2_ films could be easily controlled by the adjustment of the polymerization rate, the initial composition of component, and the type of monomers.

In principle, macroscopic domains are developed if solidification of the inorganic polymeric phase is produced after phase separation, which produces the pores. The phase separation process is induced by the strong interactions between the inorganic oligomers and the organic polymer, which repel the solvent. This results in micronic solvent droplets, which are the actual pore templates. If solidification is produced before phase separation, films with no apparent macropores are obtained. Film fluidity is required during the drying process to favor rearrangement of the nanobuilding units (in this case, Ti-oxo clusters) for macropore formation. It is also important to avoid rapid drying to permit mobility of the oligomers, to produce higher condensation grades, and to avoid flaw formation. This “race towards size-controlled pores” is similar to what is found in mesoporous thin films produced by evaporation-induced self-assembly.

Some achievements were made in previous studies, such as the successful preparation of TiO_2_ thin films using different methods and the study of film quality under different experimental conditions. However, there are many defects in TiO_2_ anode films in DSC research. Firstly, there are many grain boundaries between nanoparticles in nanocrystalline films, and the grain boundaries hinder the transport of electrons in the films. The probability of electron recombination by oxidized electrolytes also increases, which is not conducive to the effective collection of electrons. Secondly, the direction of electron transport in the anode film with a one-dimensional nanorod or nanotube structure is the same, and the barrier of the grain boundary is small. However, this structure easily vanishes from the substrate, and there is little room to improve the specific surface area of the film. Finally, the preparation process of multistage porous films is complex and takes a long time, and most of them produce small holes or mesoporous structures. These disadvantages are not conducive to the penetration of dyes and electrolytes. Therefore, these factors inhibit the improvement of DSC performance.

Aiming at the current research status and existing problems of TiO_2_ photoanode film in DSC, in this paper, the method of photopolymerization-induced phase separation is used to introduce a suitable monomer and photoinitiator. The formation of an organic polymer phase and TiO_2_ oligomer phase in the film is controlled by adjusting the speed of the hydrolysis reaction, polycondensation reaction, and polymerization reaction in the sol. Finally, TiO_2_ thin films with a three-dimensional continuous network structure were prepared. The film has the characteristics of a large specific surface area, a rich skeleton structure, and a large internal pore volume. When this film is used as the photoanode film of DSC, it is conducive to dye adsorption, rapid electron transport, and electrolyte penetration. Finally, the structure of DSC based on a three-dimensional continuous network TiO_2_ anode film is improved, and the improved method and corresponding mechanism were studied to further improve the energy conversion efficiency of DSC. In addition to the intrinsic photocatalytic activity of TiO_2_, which is directly related with its crystal properties, the structural properties of the porous TiO_2_ catalyst, such as its surface area, porosity, and pore size and distribution, are also of importance because of their potential role in enhancing the light absorbance of the TiO_2_ catalyst and the accessibility of reactants to the active catalytic sites. An interesting method to fabricate highly porous materials with the desired pore structure and size for target-specific applications is the use of amphiphilic organic molecules such as surfactants and block copolymers as pore directing agents in sol-gel methods [8,9,10,11,12]. Ionic surfactants such as alkyl phosphate, dodecylamine, and cetyltrimethylammonium chloride were initially used in the synthesis due to the strong and well-organized incorporation of a titania inorganic framework onto surfactant micelles by electrostatic interactions [9,11,12]. However, the use of ionic surfactants showed limited potential for such applications since the strong electrostatic binding force makes it difficult to remove the templates completely using extraction methods or even heat treatment at high temperatures. Common sol-gel methods employing the direct addition of water molecules in a sol can lead to the immediate precipitation of amorphous particles with an uncontrolled structure due to the rapid hydrolysis and condensation reactions.

## 2. Preparation and Characterization of Three-Dimensional Continuous Web-like TiO_2_ Thin Film

In order to prepare TiO_2_ thin films, we used absolute ethanol (EtOH) produced by Beijing chemical plant, tetrabutyl titanate (TTB), N, N-dimethylformamide (DMF), nitric acid (HNO3), and polyvinylpyrrolidone K30 (PVP) from Beijing Chemical Reagent Co., Ltd. (Beijing, China) and propylated (3) glycerol triacrylate (POGTA) from Tianjin Tianjiao chemical plant (Tianjin, China). We used the Burnett ultra-pure water system XYE2-10-H of Beijing Xiangshunyuan Technology Co., Ltd. (Beijing, China), the low-voltage mercury lamp (18 W, λmax = 254 nm) of Beijing electric light source Research Institute (Beijing, China), the vertical puller TL0.01 of Shenyang kejing Equipment Manufacturing Co., Ltd. (Shenyang, China), and the ultraviolet irradiator of the photoelectric instrument factory UV-A/B of Beijing Normal University (Beijing, China).

The TiO_2_ films with a three-dimensional web-like porous structure were prepared using the photo polymerization-induced phase separation method. Then, characterization methods including SEM, TEM, XRD, and UV-vis were used to conduct in-depth research on the obtained films.

The TiO_2_ precursor sol was first prepared. Under the condition of vigorous stirring, a certain amount of TTB was added to the mixed solution of EtOH and DMF, which was then stirred for 10 min. Subsequently, a mixture of nitric acid and deionized water was added dropwise while stirring under ice-water bath conditions so that the sol was prepared by slow hydrolysis and polycondensation. Then, the mixture was stirred for another 15 min. After removing the ice-water bath, (POGTA) was added. After stirring evenly, the photoinitiator benzoin dimethyl ether (Irgacure651) was added followed by stirring for 10 min. Finally, PVP was added and stirred until it dissolved. 

The molar ratio of the added amount of drugs was TTB: EtOH: DMF: H_2_O: HNO_3_: POGTA = 1:8:4:3:0.5:0.7. In addition, the amount of initiator benzoin dimethyl ether added was calculated based on 1.5 wt% of the mass of the light monomer POGTA. The amount of polymer PVP added was calculated as 2% of the total mass of the prepared sol.

**Figure 1:**
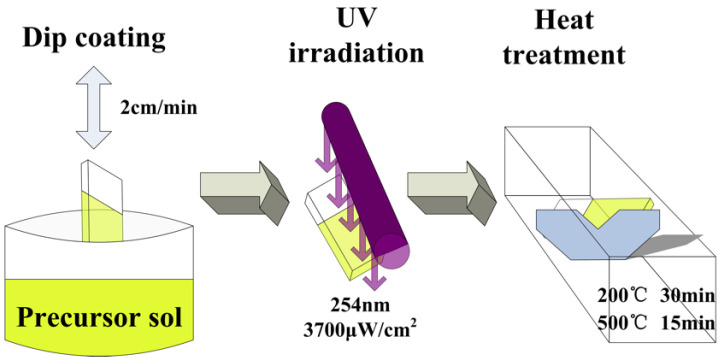
Schematic diagram of the preparation process of TiO_2_ film.

After the sol was prepared, a cleaned transparent conductive glass FTO was used as the substrate, and the vertical pulling machine was used to pull the film from the prepared sol. The pulling rate was 2 cm/min. Then, the film was quickly transferred to a UV lamp for 10 min of UV irradiation to induce monomer polymerization. Finally, the film was transferred to a box-type resistance furnace for heat treatment, which was composed of 200 °C heat treatment for 30 min followed by 500 °C heat treatment for 10 min. After these processes, the TiO_2_ film was obtained. The whole process is shown in Figure 1.

## 3. Analysis of Thin Film Morphology

**Figure 2:**
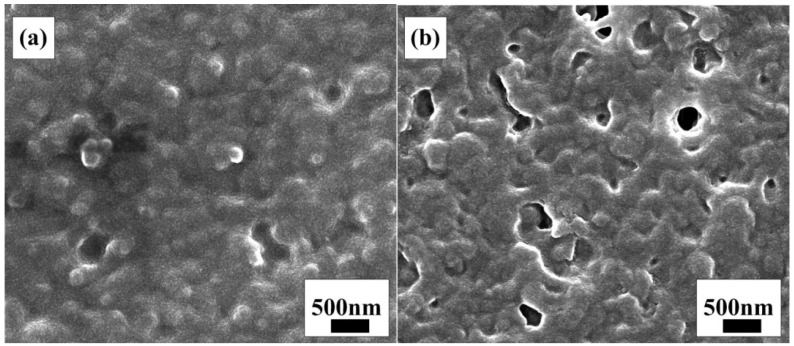
SEM images of the thin film at different stages: (**a**) TiO_2_ gel thin film before UV light irradiation; (**b**) TiO_2_ gel thin film after UV light irradiation.

Figure 2 shows the SEM results illustrating the variation in the thin film morphology (FEI Hong Kong Limited, Hong Kong, FEI Quanta200F field emission environmental scanning electron microscope, acceleration voltage 30 kV). Figure 2a is the SEM image of the naturally dried thin film without UV light irradiation, which shows that the thin film is a rough dense film formed by small particles accumulation without a pore structure on the thin film surface. Figure 2b shows the thin film morphology after UV light irradiation, which shows the partial formation of a pore structure and that the thin film is more uneven. This indicates that under the irradiation of UV energy, the substances in the sol react.

**Figure 3:**
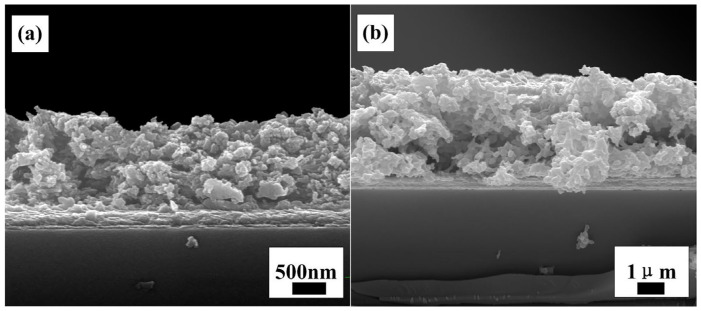
SEM images of the cross-sections of TiO_2_ thin films prepared by pulling: (**a**) one time; (**b**) seven times.

Figure 3a,b are the cross-sectional morphology images of TiO_2_ thin films prepared by pulling one time and seven times with the pulling coating process, respectively, using FTO as the substrate and transparent tape to control the thin film area. From the rulers in the figures, it can be seen that the film thickness increases with the increase in the number of coatings. The thicknesses of thin films in Figure 3a and Figure 3b are 1.3 μm and 3.3 μm, respectively.

It can also be seen from the cross-sectional morphologies that the cross-section of the thin film is uneven, which further illustrates that the skeleton of the film grows three-dimensionally in space. After multiple coatings, there are still a large number of pores in the thin film. This is conducive to the adsorption of dyes and the rapid penetration of electrolytes in the film, thereby increasing the light absorption and electron transfer speed and thus improving the performance of the battery.

## 4. TEM Characterization and Analysis

**Figure 4:**
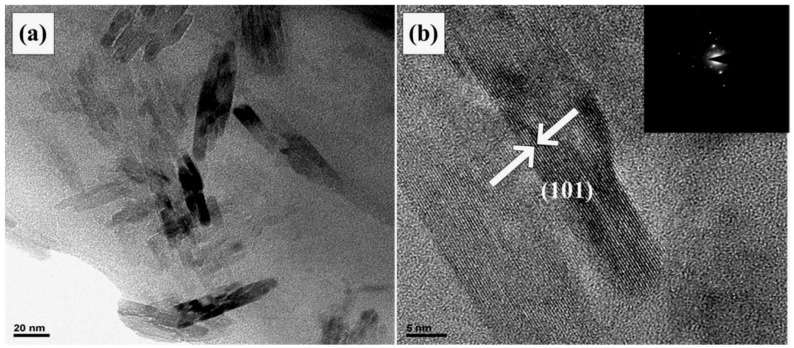
(**a**) TEM image; (**b**) HRTEM image of the web-like TiO_2_ thin film sample.

A powder sample of the prepared TiO_2_ film for TEM analysis to further study the microscopic composition was obtained by scraping the thin film from the substrate with a blade (FEI Hong Kong Co., Ltd., Hong Kong, FEI Tecnai G2 F20 field emission high resolution transmission electron microscope, acceleration voltage 200 kV). The results are shown in Figure 4. The ordinary TEM image Figure 4a shows that the film is composed of short nanorods. The average length of the short rods is 25 nm and the average particle size is 8 nm. Figure 4b is the high-resolution transmission electron microscope (HRTEM) image, which shows clear lattice fringes, with a fringe spacing of 0.3528 nm, which is consistent with the (101) crystal plane spacing of the TiO_2_ anatase single crystal phase. The inset in Figure 4b is the electron diffraction pattern of a selected area, in which only diffraction spots corresponding to the TiO_2_ anatase single crystal phase were obtained. It indicates that there were only nanoparticles of the TiO_2_ anatase single crystal phase in the selected area.

## 5. XRD Characterization and Analysis

**Figure 5:**
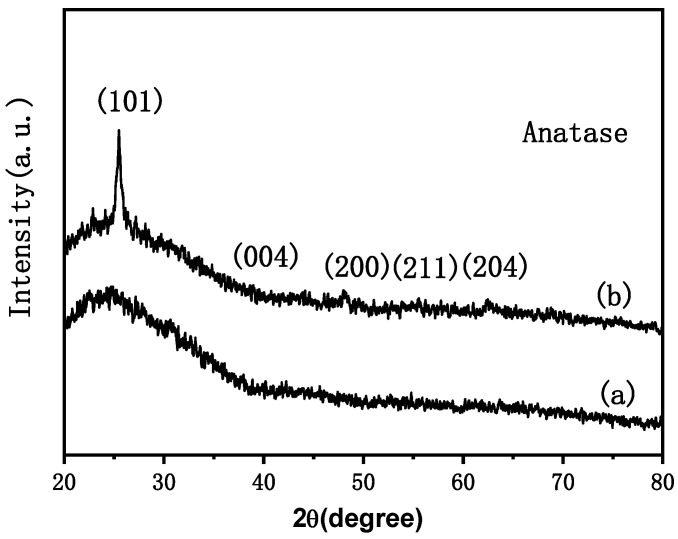
XRD spectra of the web-like TiO_2_ thin film: (a) before heat treatment; (b) prepared by pulling seven times.

Figure 5 (curves a,b) show the XRD analysis of the web-like TiO_2_ thin film before heat treatment and prepared by pulling seven times, respectively (Karlsruhe, Germany, Bruker D8 Advance X-ray diffractometer, Cu Kα ray, λ = 1.54 Å, tube voltage 40 kV, tube current 40 mA, scanning range: 20–80°). No obvious diffraction peak was observed on the diffraction spectrum of the single-layer thin film with only a bump near 25.3°. This might be because the sample was too thin or the scanning speed during the test was too fast, causing the count to be too low. There were obvious characteristic diffraction peaks at 25.3°, 37.8°, 48.1°, 54.5°, and 63.3° of the diffraction spectrum of the seven-layer thin film. According to the standard card (JCPDS, 21–1272) [41], these peaks correspond to the locations of the characteristic diffraction peaks of the TiO_2_ anatase phase, which represent the (101), (004), (200), (211), and (204) crystal planes, respectively.

It can be learned from the figure that the diffraction peak intensity of the seven-layer thin film is stronger than that of a single-layer thin film. Therefore, the crystallinity of the film obtained by multiple coating processes improved, and it was still composed of the typical anatase phase.

## 6. Thermogravimetric Differential Thermal Analysis

**Figure 6:**
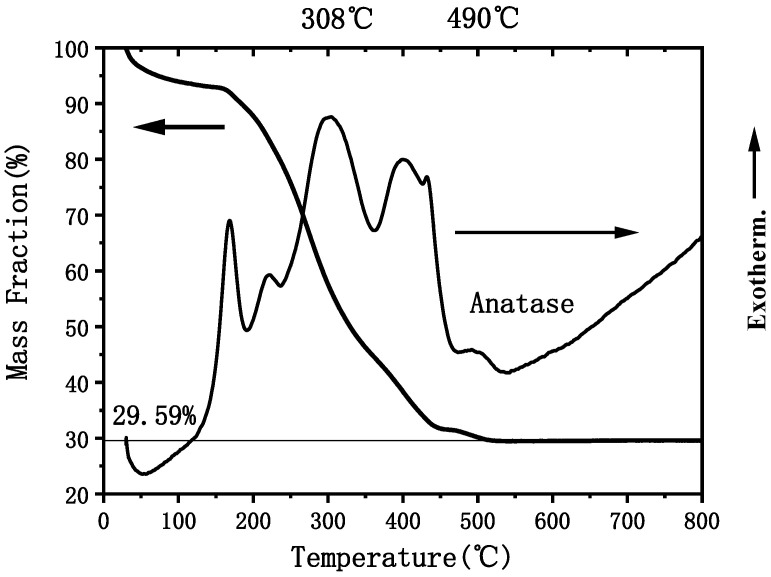
Differential heat-thermogravimetry curve of the sample scraped from the TiO_2_ gel thin film.

Figure 6 shows that thermogravimetric-differential thermal analysis results of the UV irradiated thin film scraped from the substrate using a blade (Selbu, Germany, NETZSCH STA409PC synchronous thermal analyzer, temperature range: 20~1200 °C; sensitivity: 4–4.5 μV/mW). There was an endothermic peak around 70 °C, which was caused by the evaporation of the water absorbed by the ethanol solvent and TiO_2_. Correspondingly, there was an obvious weight loss on the thermogravimetric curve, with a weight loss of about 8%. The film showed two small exothermic peaks near 167 and 220 °C, which may be due to the combustion exotherm of the remaining chemically adsorbed organic solvents and some small molecular organic compounds in the film. Correspondingly, the thermogravimetric curve was decreased by about 11%. There were two large exothermic peaks near 308 °C and 402 °C, which may be due to the combustion exotherm of some high molecular organic compounds, such as PVP, and organic monomer polymers. The thermogravimetric curve also showed a corresponding significant decrease, which was decreased by about 50%. There was a small exothermic peak near 490 °C, with the weight of the film being basically unchanged. Therefore, this exothermic peak was due to the crystallization of TiO_2_, which was caused by the transformation from amorphous to crystalline state. In addition, Figure 6 shows that the small peaks were caused by the burning of organic PVP, monomer, organic solvent, water, etc.

It can be seen that after UV light irradiation, the film contained a lot of organic matter, which was burnt out between 150 °C and 490 °C, resulting in a weight loss of 70.41%. The sample weight remained constant after 500 °C, indicating that the organic matter could be completely removed. Therefore, it is appropriate to set the final heat treatment temperature for film preparation to 500 °C.

## 7. Analysis of the Influence of POGTA Amount

The content of POGTA has a great influence on the film morphology and plays a key role in the formation of a three-dimensional continuous web-like structure. There are many double bonds in the structure of POGTA, contributing to its good spatial ductility. When the POGTA in the sol reaches a certain content, the organic polymer phase grows in space in the gel film after the polymerization reaction was completed. Then, the remaining TiO_2_ oligomer phase presents a three-dimensional web-like structure after the heat treatment.

**Figure 7:**
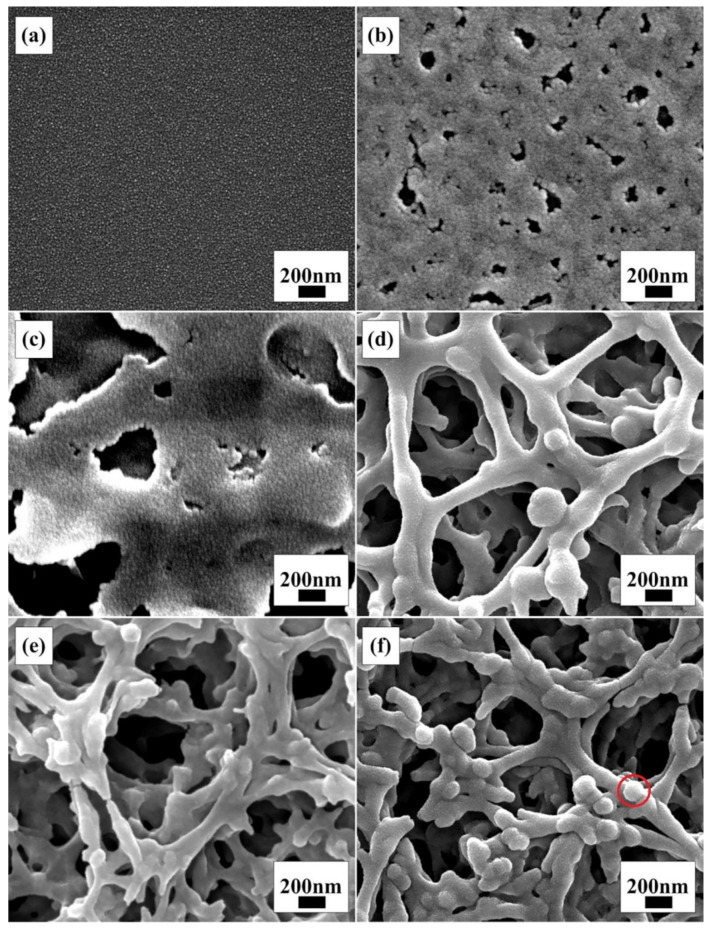
SEM images of seven-layer films prepared with different molar ratio values of POGTA/TTB: (**a**) the molar ratio of POGTA/TTB was 0; (**b**) the molar ratio of POGTA/TTB was 0.2; (**c**) the molar ratio of POGTA/TTB was 0.3; (**d**) the molar ratio of POGTA/TTB was 0.6; (**e**) the molar ratio of POGTA/TTB was 0.7; (**f**) the molar ratio of POGTA/TTB was 0.8.

Figure 7a–f are SEM images of the seven-layer films prepared when the amount of POGTA in the sol was adjusted.

After coating multiple times, the morphological characteristics of the film of each ratio were similar to the corresponding single-layer film. When POGTA/TTB = 0, the film was dense. When POGTA/TTB = 0.2, the film presented a porous structure. When POGTA/TTB = 0.3, the surface morphology remained a simple single-layer skeleton structure. When POGTA/TTB increased to 0.6 and above, due to the larger average pore size, the upper skeleton would not completely block the pores of the lower layer, and thus the porosity on the film surface was very considerable. The mutual coverage between different layers of film made the network structure of the film more abundant.

**Figure 8:**
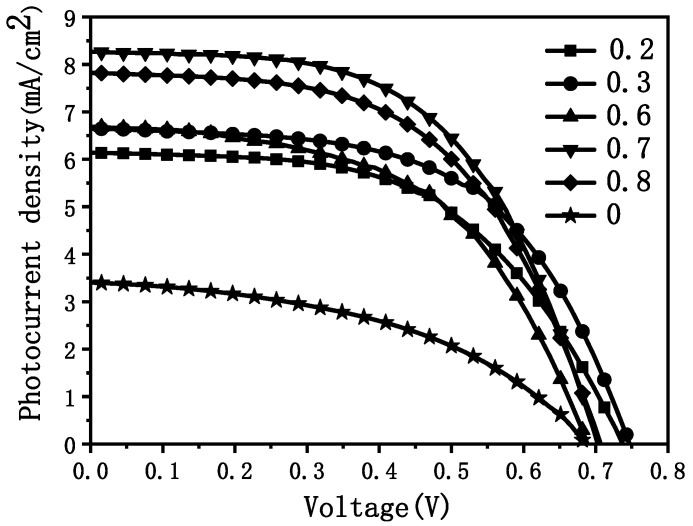
I–V test curves of seven-layer thin film anode DSC when the molar ratio POGTA/TTB = 0, 0.2, 0.3, 0.6, 0.7, 0.8.

After assembling the DSC, the corresponding I–V test results are shown in Figure 8. Table 1 is the corresponding battery performance parameters. As the POGTA content increased, the short-circuit current density was increased significantly, while the open-circuit voltage and fill factor also increased. When POGTA/TTB = 0.8, the short-circuit current density and FF started to decrease. The final energy conversion efficiency reached the maximum when POGTA/TTB = 0.7.

Combined with the SEM analysis of the anode thin film, it can be learned that, initially, with the gradual increase in the POGTA content, the web-like structure became abundant and the surface porosity was increased. It is beneficial for the dye to form single-layer adsorption on the surface of the skeleton and the electrolyte to quickly penetrate into the inside of the film, thereby contributing to the rapid generation and transport of photo-generated electrons and thus increasing the short-circuit current density and energy conversion efficiency. However, when POGTA/TTB = 0.8, even though there were some small protrusions on the skeleton of the film, like the part in the red circle in the picture, which helped to increase the surface area of the film to some degree, the skeleton density remained low. As a result, the transport performance of electrons inside the film might decrease, so the short-circuit current density of the assembled DSC was smaller than when POGTA/TTB = 0.7, and the energy conversion efficiency was also reduced. Therefore, the molar ratio POGTA/TTB was selected to be 0.7.

## 8. Study on the Effect of TiO_2_ Surface-Modification Layer on DSC Performance

During the electron transport process of DSC, the electrons on the FTO react with the oxidized electrolyte in the electrolyte. Electrons cannot be collected effectively, which is an important factor that inhibits the improvement of DSC efficiency. The three-dimensional continuous web-like structure of the film has large pores, which is very conducive to the penetration of electrolytes into the inside of the film. However, it also increases the probability of direct contact between the electrolyte and the surface of the FTO, resulting in an increase in the adverse effects of this composite reaction. Therefore, effectively inhibiting the composite reaction is one of the important ways to improve the effective charge collection efficiency. In this paper, the TiCl_4_ hydrolysis method was used to pretreat the surface of the FTO substrate. Thus, a dense TiO_2_ barrier layer was added to the surface of the FTO to study the influence of the TiO_2_ as a surface-modification layer on the DSC properties using the anodic film immersion method. By adding a layer of material (TiCl_4_) with a higher energy level than TiO_2_ onto the surface of the TiO_2_ anode film, the electron transfer in the TiO_2_ conduction band can be reduced. The material can effectively inhibit the TiO_2_/dye/electrolyte interface composite reaction, so as to effectively collect electrons. Therefore, the short-circuit current density and energy conversion efficiency of DSC are improved.

**Figure 9:**
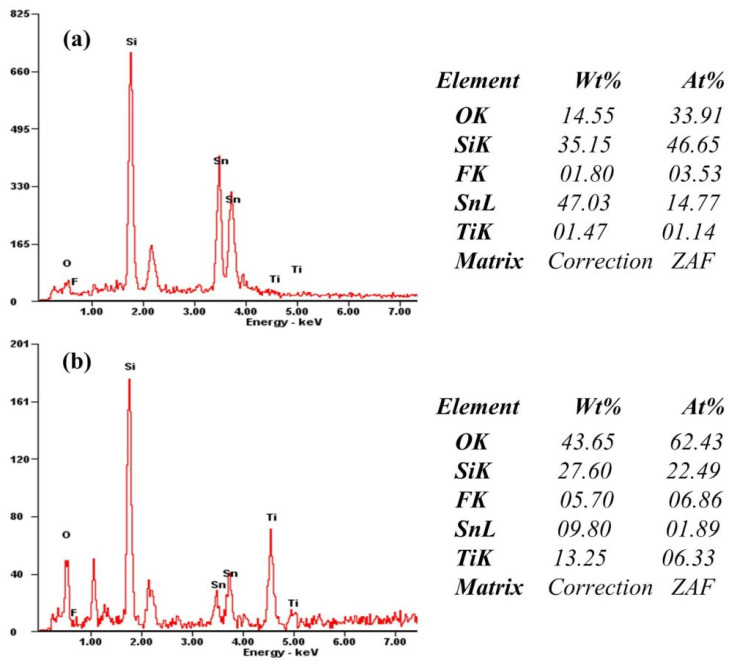
Front EDS spectrum (**a**) and cross-section EDS spectrum (**b**) of the FTO substrate after adding barrier layer.

Figure 9a,b show the EDS energy spectrum analysis of the front and cross-section of the FTO substrate after adding the barrier layer onto the surface of FTO substrate. The right side of the picture shows the mass percentage and atomic weight percentage of the corresponding elements. From the EDS energy spectrum, it can be determined that there is Ti element in the FTO substrate after adding the barrier layer.

**Figure 10:**
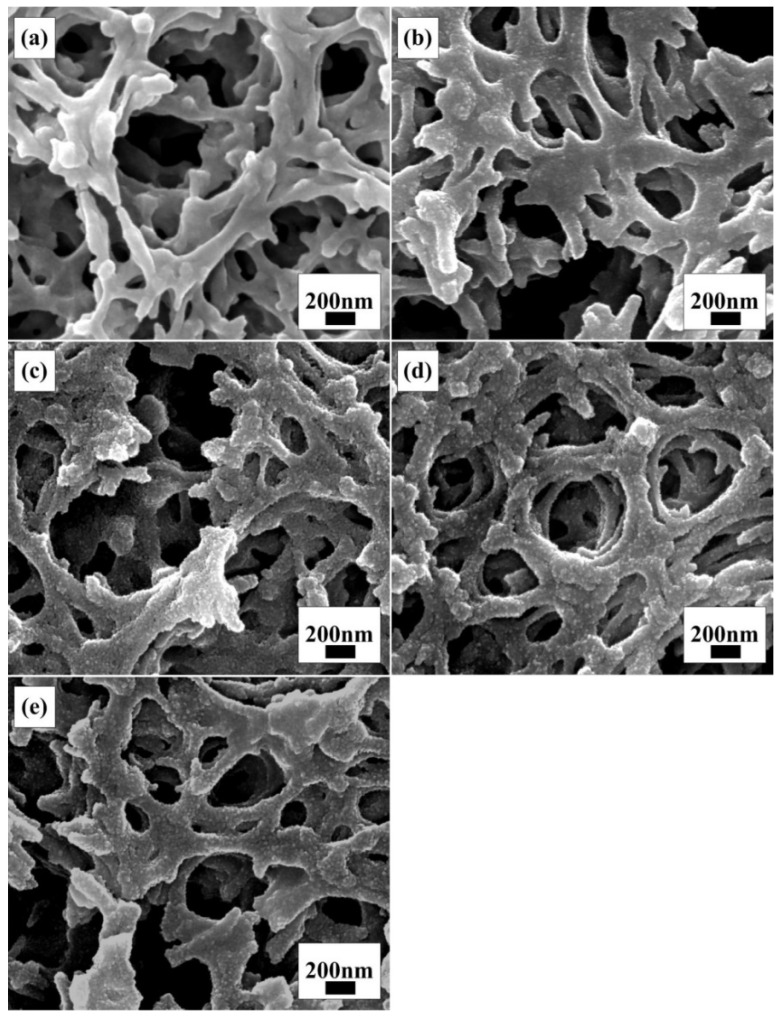
Surface SEM images of anode films after immersion in 40 mM TiCl_4_ aqueous solution at 70 °C for different times: (**a**) 0 min; (**b**) 15 min; (**c**) 30 min; (**d**) 60 min; (**e**) 75 min.

Figure 10 shows the surface SEM of the anode film modified by immersing it in a 40 mM TiCl_4_ aqueous solution at 70 °C for different times. It can be seen that, compared with unmodified film (Figure 10a), the skeleton of the modified film was evenly covered with a layer of particles with a particle size of about 10 nm. In addition, with the extension of the immersion time, the number of particles increased, which can increase the surface roughness of the film to a certain extent.

**Figure 11:**
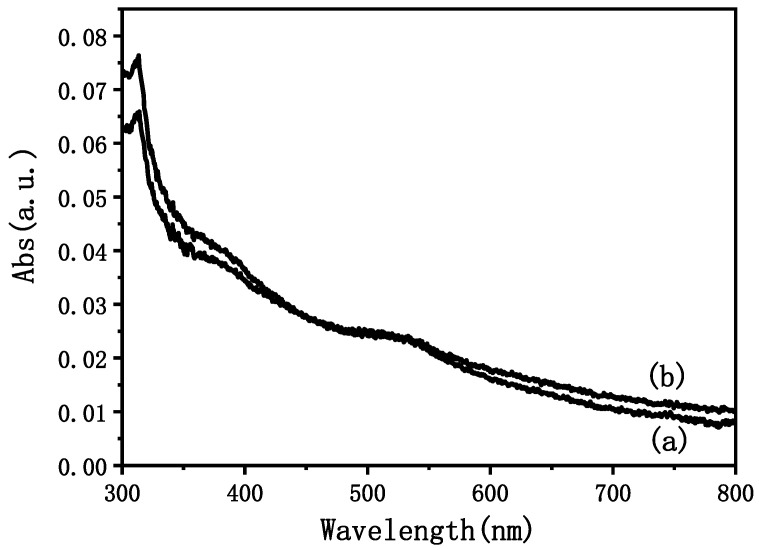
The absorbance curve of the dye-adsorbed film after being immersed in NaOH ethanol solution for 40 min: (a) unmodified anode film; (b) anode film immersed in 40 mM TiCl_4_ aqueous solution at 70 °C for 30 min.

In order to study the change in N719, the modified and unmodified TiO_2_ films that had been loaded with N719 were immersed in 0.1 moL NaOH ethanol solution, and N719 was integrated into the NaOH solution. We characterized the solution using the UV absorption spectrum, and obtained the change in N719 load using the absorbance. Figure 11 shows the absorbance test results of the resulting solutions. The absorbance value of the film before modification at 520 nm was 0.02408, from which the concentration of N719 ((C_58_H_86_O_8_N_8_S_2_Ru, RuL_2_ (NCS)_2_:2TBA, 5 × 10^−4^ M)) in the solution was calculated to be 11.412 × 10^−7^ M. The corresponding absorbance value of the modified film was 0.02460, and the calculated concentration of N719 in the solution was 11.659 × 10^−7^ M. This indicates that after being soaked in a 40 mM TiCl_4_ aqueous solution at 70 °C for 30 min, the dye adsorption capacity of the anode film was enhanced.

**Figure 12:**
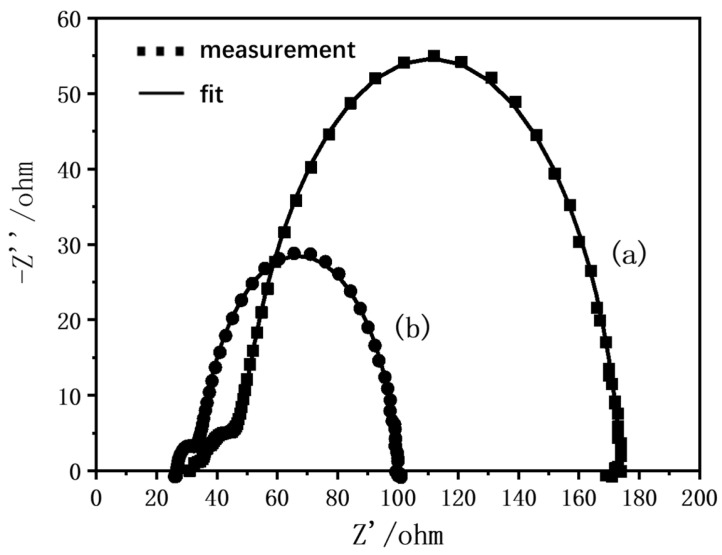
EIS results of DSC after assembly of anode films: (a) without modification; (b) modified by soaking in 40 mM TiCl_4_ aqueous solution at 70 °C for 30 min.

In this paper, the CHI electrochemical workstation from Shanghai Chenhua Instrument Co., Ltd. (Shanghai, China) was used for the DSC impedance analysis. The initial potential was the DSC stable open-circuit potential, the test frequency range was 10^6^–10^−2^ Hz, and the disturbance amplitude was v. Figure 12 (curves a,b) show the Nyquist diagrams obtained from the EIS test at a standard light intensity before and after adding the TiO_2_-modified layer to the DSC at 25 °C. EIS corresponds to the equivalent circuit diagram of DSC. The point corresponds to the result of the original measurement data, and the curve corresponds to the fitted result. Table 2 shows the corresponding electron transport dynamics microscopic parameters. In the table, R_s_ represents the series resistance at FTO, R_pt_ represents the charge transfer resistance at the interface between the Pt counter electrode and electrolyte, R_rec_ represents the transfer resistance of electrons at the interface of the anode film/dye/electrolyte, and R_dc_ represents the sum of RS, RPT, and rrec. *K_eff_* represents the effective recombination rate at the anode/dye/electrolyte interface, *τ_eff_* represents the effective electron lifetime at the anode film/dye/electrolyte interface, and *n_s_* represents the electron density in the TiO_2_ conduction band in a steady state. It can be seen from the data that, compared with DSC without the modification layer, the series resistance of DSC, and the charge transfer resistance of the Pt counter electrode/electrolyte interface and TiO_2_/dye/electrolyte interface were significantly reduced after the anode film was modified by soaking it in 40 mM TiCl_4_ aqueous solution at 70 °C for 30 min. This greatly reduced the total DC resistance that electronic transport needed to overcome. It shows that the small particles of TiO_2_ attached to the skeleton strengthen the connection between skeletons, which enhances the conductivity of the skeleton to electrons, and effectively reduces the obstacles to electron transport. However, the increase in the effective composite rate of the TiO_2_/dye/electrolyte interface reduces the electronic lifetime of the interface. The possible reason is that the small particles of TiO_2_ attached to the skeleton increase the specific surface area of the film, that is, the contact area with the electrolyte increases, and thus the composite rate increases. Combining the above-mentioned electron transport obstacles and electron lifetime, the electron density at the conduction band of the anode TiO_2_ increases.

**Figure 13:**
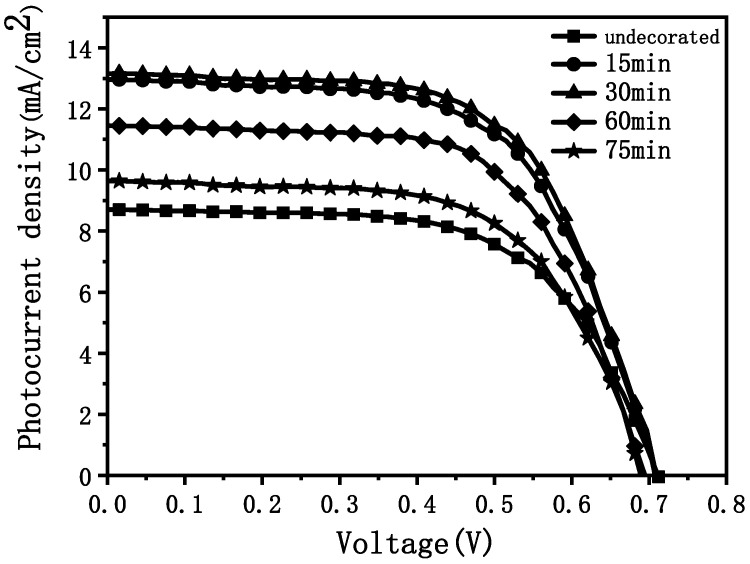
I–V curves of DSC after immersing anode film in 40 mM TiCl_4_ aqueous solution at 70 °C for different times.

Figure 13 shows the I–V test curves of the assembled DSC with anode film soaked in a 40 mM TiCl_4_ aqueous solution at 70 °C for different times for surface modification. Table 3 shows the corresponding battery performance parameter values.

**Figure 14:**
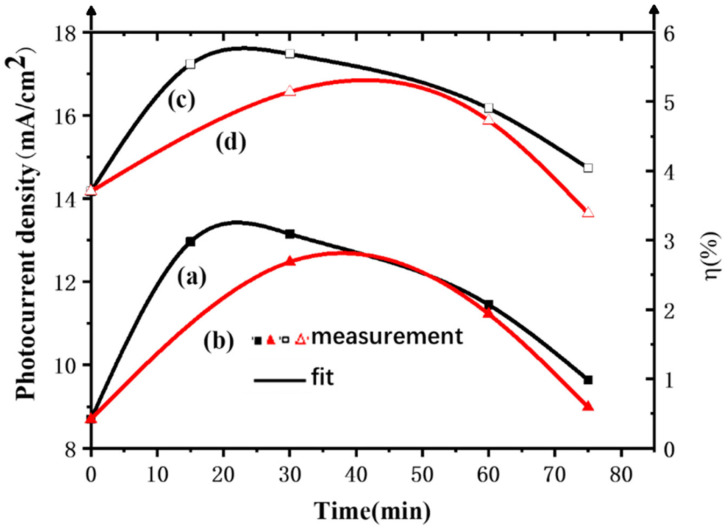
Variations in short-circuit current densities versus immersion time of DSC after surface modification with different concentrations of TiCl_4_ aqueous solution: (a) 40 mM; (b) 50 mM. Variations in energy conversion efficiencies versus immersion time of DSC after surface modification with different concentrations of TiCl_4_ aqueous solution: (c) 40 mM; (d) 50 mM.

After being modified by TiCl_4_, the open-circuit voltages were almost unchanged, while both the short-circuit current densities and FFs were increased. The final energy conversion efficiencies were also improved. Moreover, as shown in curve (a) in Figure 14, as the immersion time was prolonged, the short-circuit current density showed a trend of first increasing and then decreasing, reaching the maximum at 30 min. Further increases in the immersion time caused the short-circuit current density to decrease.

It can be seen from the above that, after being immersed in the TiCl_4_ aqueous solution, the skeleton of the film was uniformly covered by a layer of small nanoparticles, which increased the surface roughness of the film and facilitated dye adsorption. In addition, nanoparticles attached to the skeleton can strengthen the connectivity between the skeletons, so that the transport obstruction of electrons was reduced, and the FF was increased. This contributes to the collection of electrons, leading to an increased short-circuit current density. However, when the immersion time was too long, too many TiO_2_ particles were deposited, and the grain boundary barriers that had to be overcome to transport the electrons generated on the film surface to the FTO increased. Therefore, the short-circuit current density first increased and then decreased with the immersion time. As a result, the energy conversion efficiency also increased first and then decreased with the extension of the immersion time (as shown in curve (c) in Figure 14). The maximum energy conversion efficiency 5.6836% was reached when the immersion time was 30 min. When the concentration of TiCl_4_ aqueous solution increased, the rate of the TiCl_4_ hydrolysis reaction increased. Under the same soaking time, more TiO_2_ nanoparticles were attached to the mesh skeleton of the film. Compared with the unmodified film, the surface pore density of the modified film decreased significantly, which seriously affected the adsorption of dyes and the penetration of electrolytes. Therefore, under the same soaking time, the short-circuit current density of DSC soaked in 50 mM TiCl_4_ aqueous solution was lower than that of the DSC soaked in 40 mM, resulting in a low energy conversion efficiency.

## 9. Conclusions

The TiO_2_ films with a three-dimensional web-like porous structure were prepared using the photo polymerization-induced phase separation method. The film skeleton structure was composed of short nanorods of the TiO_2_ anatase phase. The average length of the short rods was 25 nm and the average particle size was 8 nm. The skeleton size was about 150 nm, and the pore size of the film was distributed between 100 nm and 1 μm. After using multiple coating processes, the morphology of the film was more uniform, and the web-like structure was more abundant. After coating for seven times, the film thickness reached 3.3 μm. Compared with a single-layer film, the crystallinity of the film was improved, which provided more active sites for dye adsorption.

The content of acidic inhibitor HNO_3_, photomonomer POGTA, and polymer PVP in the precursor sol has a great influence on the morphology and structure of the TiO_2_ film, which in turn affects the photoelectric conversion performance of DSC. It was learned in the experiments that when POGTA/TTb = 0.7, the web-like structure became abundant, and the surface pore density was high, which is most conducive to the formation of monolayer adsorption of the dye on the surface of the skeleton and the rapid penetration of electrolytes into the film. This contributes to the rapid generation and transport of photogenerated electrons, which increases the short-circuit current density and energy conversion efficiency. Compared with other experimental groups, the short-circuit current density of 8.2680 mA/cm^2^ and energy conversion efficiency of 3.2356% were the highest at POGTA/TTb = 0.7. After soaking in TiCl_4_ aqueous solution, the skeleton of the film was uniformly covered by a layer of small nanoparticles, which increased the surface roughness of the film and is beneficial to dye adsorption and the energy conversion efficiency. The maximum energy conversion efficiency of 5.6836% was reached when the immersion time was 30 min. Meanwhile, the maximum short-circuit current density of 13.1507 mA/cm^2^ was obtained. There is a large gap between the energy conversion efficiency of 5.6836% and that of the conversion efficiency of commercial solar cells (more than 20%), and further research is needed to realize mass production and use. In this paper, the methods and techniques of how to decrease the hole size and increase the specific surface area have not been explored sufficiently.

In the future, we will explore ways in which to use other materials to modify the surface of DSC based on the three-dimensional continuous web-like structure of TiO_2_ anode films, and the best preparation process. Other photomonomers will be used to prepare TiO_2_ porous film using the photo polymerization-induced phase separation method, and its application in DSC will be explored.

## Figures and Tables

**Table 1:** The performance parameters of the photoanode assembly DSC of the seven-layer TiO_2_ film prepared under different conditions of POGTA/TTB.

The Molar Ratio POGTA/TTB of the DSC Photoanode Material	Open Circuit Voltage Voc (V)	Short-Circuit Current Density Jsc (mA/cm^2^)	Filling Factor FF (%)	Energy Conversion Efficiency η (%)
0	0.6817	3.4057	45.79	1.0633
0.2	0.7272	6.1426	55.35	2.4722
0.6	0.6817	6.6902	54.45	2.4835
0.7	0.6969	8.2680	56.16	3.2356
0.8	0.6969	7.8209	55.32	3.0150

**Table 2:** Electron transport kinetics microscopic parameters of DSC before and after adding TiO_2_ particle surface-modification layer.

DSC Photo Anode Material	R_s_ (Ω)	R_pt_ (Ω)	R_rec_ (Ω)	R_dc_ (Ω)	$K_{eff}\;(s^{-1})$	$\tau_{eff}\;(s)$	$n_{s}\;(m^{-3})$
Unmodified	34.41	14.81	124.7	173.92	8.25	0.12	1.89 × 10^24^
Soaked for 30 min	26.75	7.265	66.11	100.125	10.0	0.10	2.95 × 10^24^

**Table 3:** Performance parameters of DSC assembled with anode thin film after soaking in 40 mM TiCl_4_ aqueous solution at 70 °C for different times.

DSC Photoanode Material	Open-Circuit Voltage Voc (V)	Short-Circuit Current Density Jsc (mA/cm^2^)	Filling Factor FF (%)	Energy Conversion Efficiency η (%)
Unmodified	0.7121	8.6977	59.80	3.7040
15 min	0.7121	12.9611	59.99	5.5373
30 min	0.7121	13.1507	60.69	5.6836
60 min	0.6963	11.4495	61.47	4.9012
75 min	0.6963	9.6406	60.16	4.0386
